# Videolaryngoscopy As the Primary Approach to Emergency Airway Management in Facial Trauma: A Case Report

**DOI:** 10.7759/cureus.74536

**Published:** 2024-11-26

**Authors:** Nisha Jain, Abhishek Singh

**Affiliations:** 1 Anesthesiology, Pain Medicine and Critical Care, All India Institute of Medical Sciences, New Delhi, New Delhi, IND

**Keywords:** airway management, difficult airway management, emergency airway, trauma management in maxillofacial region, video laryngoscopy (vl)

## Abstract

Managing the airway in maxillofacial trauma poses significant challenges. The distorted anatomy often complicates face mask ventilation and intubation, necessitating specialized skills in emergency settings. Successful management hinges on prompt planning and patient cooperation. Here, we describe the airway management of a bullhorn fascial injury patient requiring wound exploration and repair under general anesthesia. Timed clinical judgment and an adaptable approach ensured effective airway management using videolaryngoscopy as the primary approach in this case. In addition, we have described the probable difficulties that may be faced while managing the airway of such patients and how to deal with these challenges.

## Introduction

Effective management of maxillofacial trauma requires close collaboration among anesthesiologists, trauma surgeons, and maxillofacial surgeons [1]. Among in-hospital trauma patients, errors in managing airway and respiratory parameters are prevalent and can significantly impact morbidity and mortality rates [2]. In emergency scenarios, navigating challenging airways relies heavily on clinical judgment and expertise. By fostering teamwork, meticulous airway management planning, and adept performance, complications can be minimized [3]. Notably, the use of a videolaryngoscope for checking laryngoscopy as a prime method for managing the airway in patients with maxillofacial trauma is well established. Here, we describe their importance in managing difficult airways due to maxillofacial trauma.

## Case presentation

**Figure 1:**
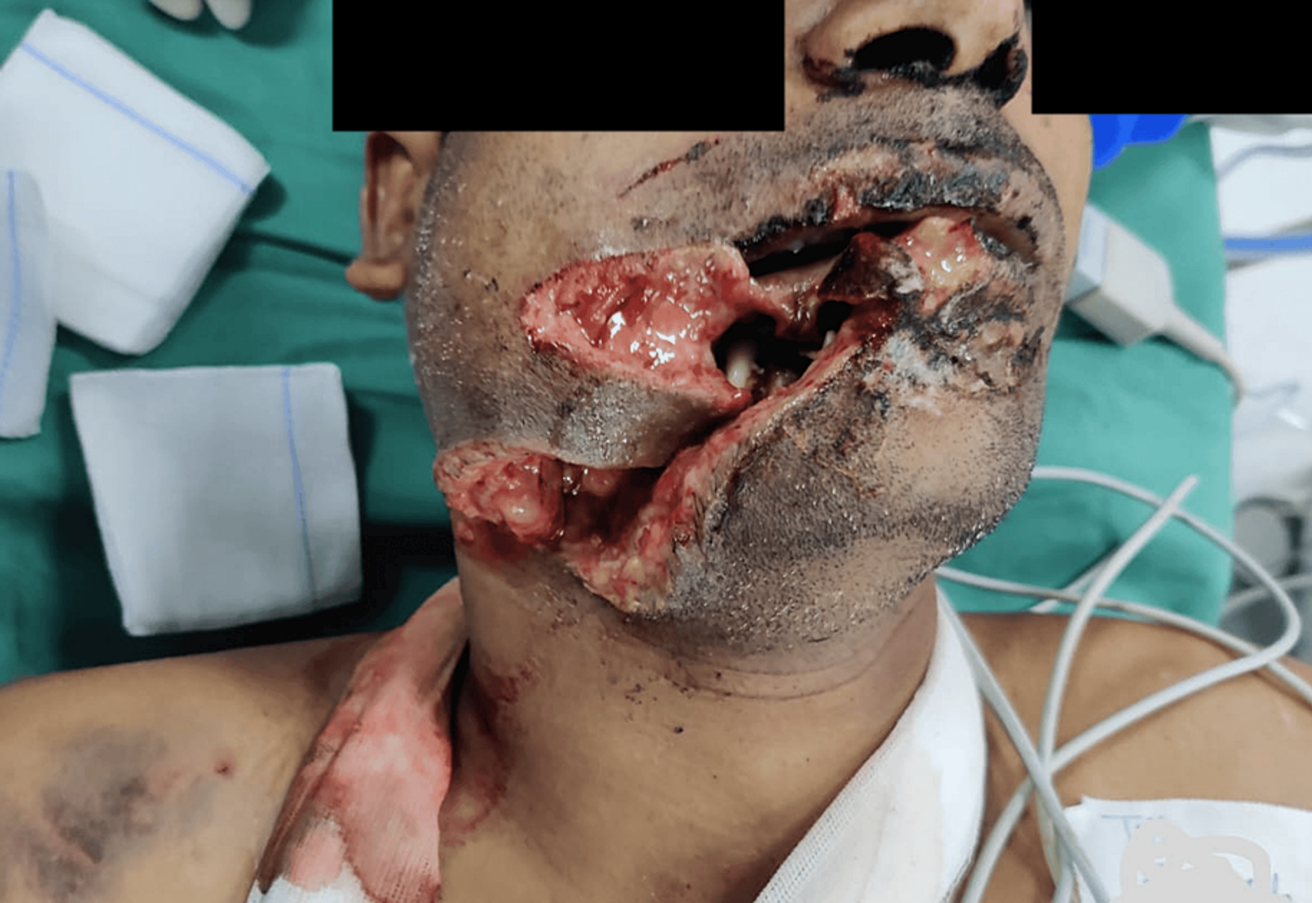
The presence of extensive soft tissue injuries on the right side of the face.

A 40-year-old man was brought to the emergency department with an alleged history of bullhorn injury to the face. Primary and secondary surveys were conducted in accordance with the advanced trauma life support guidelines. The patient was conscious, oriented, and spontaneously breathing room air. Airway examination revealed extensive soft tissue injuries along with a mandible fracture on the right side of the face (Figure 1).

The nasopharyngeal airway was placed to ensure airway patency. The respiratory pattern and rate were normal with an unremarkable auscultation. The patient was hemodynamically stable with a Glasgow coma scale score of 15/15. A decision for primary facial reconstruction under general anesthesia was made. Informed written consent was obtained, and the patient was wheeled to the operation theater (OT). His vital parameters recorded in OT were a heart rate of 110 beats/min, noninvasive blood pressure of 134/80 mm Hg, and SpO2 of 100% on room air. In view of the extensive deconstruction of the face, the preferred approach for safe management of the airway was checked laryngoscopy with videolaryngoscopy under inhalational induction, with the maintenance of spontaneous respiration followed by definitive surgery under general anesthesia. Nasal prongs were used for periprocedural oxygen supplementation.

**Figure 2:**
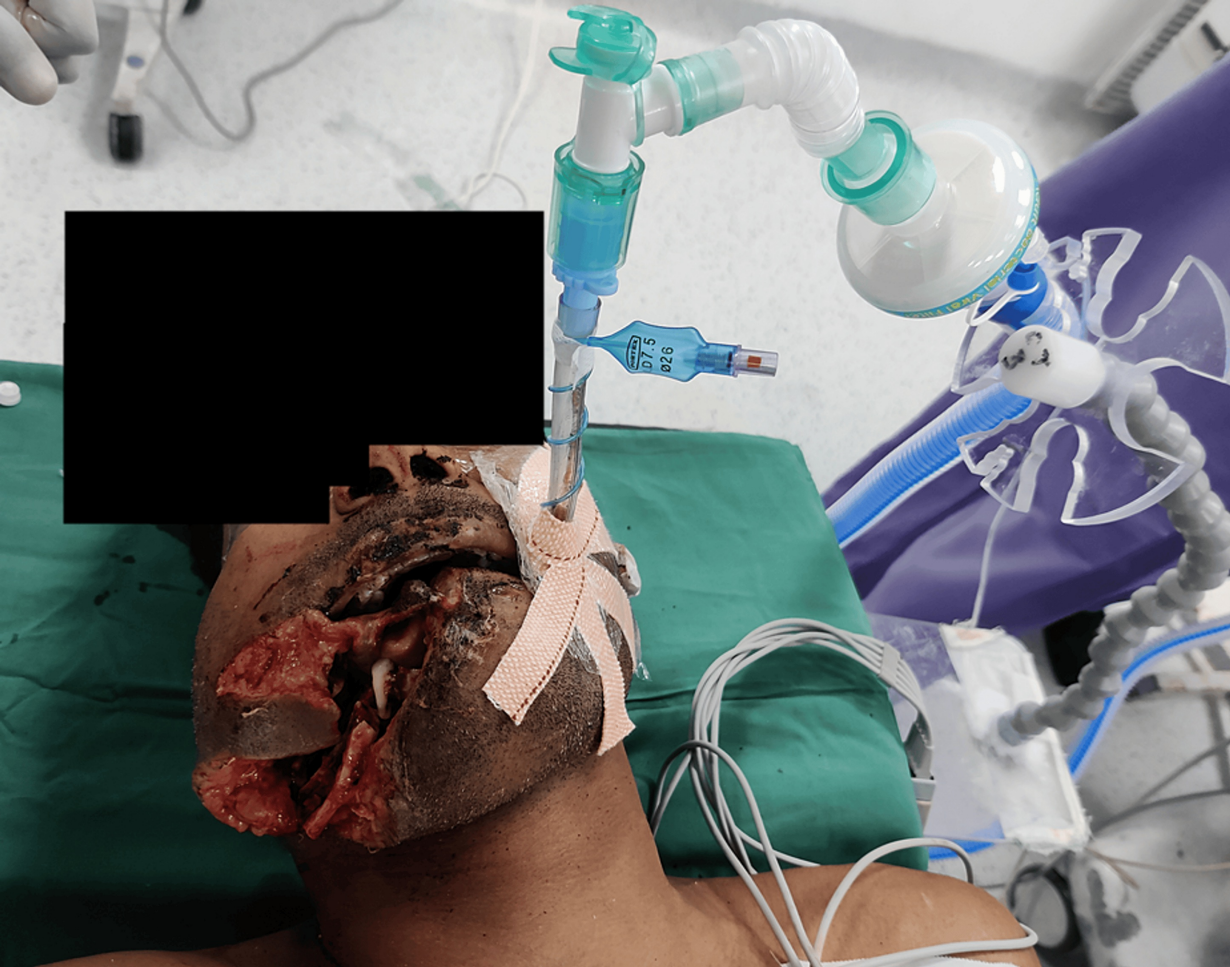
The endotracheal tube is fixed on the opposite side of the injury.

Since there was an extensive soft tissue injury, which may lead to an air leak during bag-mask ventilation, the injured part was packed with gauge pieces after removing blood clots, secretions, and mandible bone fragments. The airway was cleared by oral suction, followed by a 10% lignocaine spray for topical anesthesia of the airway. The patient was preoxygenated with 100% oxygen for 5 minutes with the help of a facemask. Anesthesia was induced with sevoflurane in 100% oxygen while maintaining spontaneous respiration. Once we achieved the minimum alveolar concentration (MAC) of 1.2, laryngoscopy was performed with a C-MAC® videolaryngoscope (Karl Storz SE & Co. KG, Tuttlingen, Germany), which helped us to visualize the vocal cords after thorough oropharyngeal suctioning. As soon as a good glottic view was obtained, fentanyl and the muscle relaxant rocuronium were administered, and after the bag-mask ventilation for 90 seconds, the trachea was intubated in a single attempt with an 8-millimeter cuffed endotracheal tube, and the position was confirmed by end-tidal carbon dioxide. For the right-side cheek surgery, the endotracheal tube was fixed on the opposite side of the face (Figure 2).

**Figure 3:**
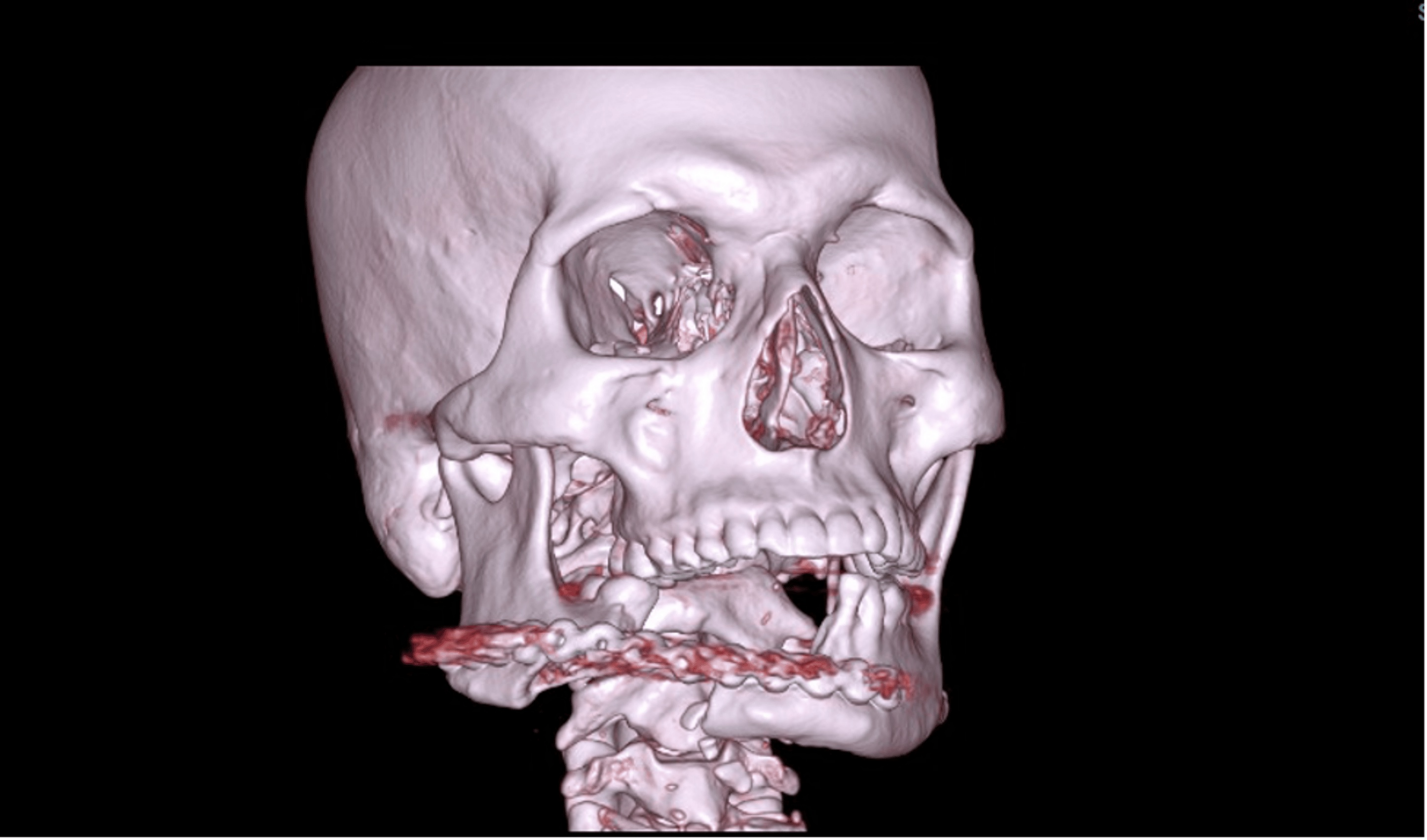
The 3D reconstruction of the face shows the implant in situ.

Surgeons proceeded with debridement and repair. The postoperative course of the patient was uneventful (Figure 3).

## Discussion

It is evident that maintaining a patent airway in cases of extensive facial injuries is of paramount importance because of the risk of compromised airways from tissue injury, edema, and fractures. The initial focus should be on preventing posterior airway collapse by manually repositioning fractured mandibular segments and managing the tongue base. Temporary measures such as nasopharyngeal and oropharyngeal airways can provide immediate relief, but the ultimate goal is to secure the airway through endotracheal intubation, despite the challenges posed by tissue damage, bleeding, and patient positioning [4].

In emergency situations, a thorough and rapid assessment of the airway, combined with the evaluation of the patient’s hemodynamic status, is crucial for determining the appropriate airway management plan. Various devices and strategies have been developed to enhance visualization of the glottis, aiding in successful intubation and ensuring adequate oxygenation and ventilation for patient survival. In cases where traditional methods of intubation are challenging because of limited space or risk of further damage, alternative techniques such as intubating laryngeal mask airways or supraglottic airway devices may be considered. However, it is important to note that the insertion of these devices can still pose difficulties and carry a risk of aspiration. In situations where traditional intubation methods are not feasible or unsuccessful, direct access to the trachea through cricothyroidotomy or tracheotomy may be necessary [1]. However, these procedures are invasive and require specialized training.

Fiberoptic bronchoscopy (FOB) is considered a standard tool for all such difficult airway management, but its utility may be limited in cases of maxillofacial trauma due to obscured vision caused by blood, secretions, and debris. In addition, trauma patients may not be cooperative enough to undergo awake FOB-guided intubation [5]. Emerging techniques such as videolaryngoscopy-assisted fiberoptic intubation and videolaryngoscopy in the high Fowler's position offer potential alternatives for managing difficult airways in patients with maxillofacial trauma [6,7]. These methods improve the visualization of airway structures, facilitating safer and more effective intubation in challenging cases. In cases of extensive maxillofacial trauma, surgical airway management may be necessary, but it was not required in our case because there were no signs of respiratory distress and prolonged postoperative intubation was not anticipated.

## Conclusions

The management of a traumatic difficult airway requires a comprehensive approach involving thorough assessment, close-loop communication, critical decision-making, and meticulous preparation. By adapting anesthesia techniques to ensure patient comfort and compliance, while also prioritizing safety and effectiveness, healthcare providers can mitigate the potential for catastrophic outcomes. Successful and timely interventions in such cases hinge on the ability to navigate the complexities of airway management with skill and precision.
